# Non-Targeted Metabolomics Analysis of Golden Retriever Muscular Dystrophy-Affected Muscles Reveals Alterations in Arginine and Proline Metabolism, and Elevations in Glutamic and Oleic Acid In Vivo

**DOI:** 10.3390/metabo7030038

**Published:** 2017-07-29

**Authors:** Muhammad Abdullah, Joe N. Kornegay, Aubree Honcoop, Traci L. Parry, Cynthia J. Balog-Alvarez, Sara K. O’Neal, James R. Bain, Michael J. Muehlbauer, Christopher B. Newgard, Cam Patterson, Monte S. Willis

**Affiliations:** 1 Department of Biochemistry, QuaidiAzam University, 45320 Islamabad, Pakistan; abdlakhn@yahoo.com; 2 Department of Pathology & Laboratory Medicine, University of North Carolina, Chapel Hill, NC 27599-7525, USA; traci_parry@med.unc.edu; 3 McAllister Heart Institute, University of North Carolina, Chapel Hill, NC 27599-7126, USA; 4 Department of Veterinary Integrative Biosciences, College of Veterinary Medicine and Biomedical Sciences, Texas A&M University, College Station, TX 77843, USA; JKornegay@cvm.tamu.edu (J.N.K.); cbalog@tamu.edu (C.J.B.-A.); 5 Toxicology Curriculum, University of North Carolina, Chapel Hill, NC 27599, USA; n18d478@email.unc.edu; 6 Sarah W. Stedman Nutrition and Metabolism Center, Duke Molecular Physiology Institute, Duke University Medical Center, Durham, NC 27708, USA; sara.oneal@duke.edu (S.K.O.’N.); james.bain@duke.edu (J.R.B.); michael.muehlbauer@duke.edu (M.J.M.); newga002@mc.duke.edu (C.B.N.); 7 Division of Endocrinology, Metabolism, and Nutrition, Department of Medicine, Duke University Medical Center, Durham, NC 27703, USA; 8 Presbyterian Hospital/Weill-Cornell Medical Center, New York, NY 10065, USA; cpatters@nyp.org; 9 Department of Pharmacology, University of North Carolina, Chapel Hill, NC 27599, USA

**Keywords:** Duchenne muscular dystrophy, golden retriever muscular dystrophy, skeletal muscle, metabolism, non-targeted metabolomics

## Abstract

Background: Like Duchenne muscular dystrophy (DMD), the Golden Retriever Muscular Dystrophy (GRMD) dog model of DMD is characterized by muscle necrosis, progressive paralysis, and pseudohypertrophy in specific skeletal muscles. This severe GRMD phenotype includes moderate atrophy of the biceps femoris (BF) as compared to unaffected normal dogs, while the long digital extensor (LDE), which functions to flex the tibiotarsal joint and serves as a digital extensor, undergoes the most pronounced atrophy. A recent microarray analysis of GRMD identified alterations in genes associated with lipid metabolism and energy production. Methods: We, therefore, undertook a non-targeted metabolomics analysis of the milder/earlier stage disease GRMD BF muscle versus the more severe/chronic LDE using GC-MS to identify underlying metabolic defects specific for affected GRMD skeletal muscle. Results: Untargeted metabolomics analysis of moderately-affected GRMD muscle (BF) identified eight significantly altered metabolites, including significantly decreased stearamide (0.23-fold of controls, p = 2.89 × 10^−3^), carnosine (0.40-fold of controls, p = 1.88 × 10^−2^), fumaric acid (0.40-fold of controls, p = 7.40 × 10^−4^), lactamide (0.33-fold of controls, p = 4.84 × 10^−2^), myoinositol-2-phosphate (0.45-fold of controls, p = 3.66 × 10^−2^), and significantly increased oleic acid (1.77-fold of controls, p = 9.27 × 10^−2^), glutamic acid (2.48-fold of controls, p = 2.63 × 10^−2^), and proline (1.73-fold of controls, p = 3.01 × 10^−2^). Pathway enrichment analysis identified significant enrichment for arginine/proline metabolism (p = 5.88 × 10^−4^, FDR 4.7 × 10^−2^), where alterations in L-glutamic acid, proline, and carnosine were found. Additionally, multiple Krebs cycle intermediates were significantly decreased (e.g., malic acid, fumaric acid, citric/isocitric acid, and succinic acid), suggesting that altered energy metabolism may be underlying the observed GRMD BF muscle dysfunction. In contrast, two pathways, inosine-5′-monophosphate (VIP Score 3.91) and 3-phosphoglyceric acid (VIP Score 3.08) mainly contributed to the LDE signature, with two metabolites (phosphoglyceric acid and inosine-5′-monophosphate) being significantly decreased. When the BF and LDE were compared, the most significant metabolite was phosphoric acid, which was significantly less in the GRMD BF compared to control and GRMD LDE groups. Conclusions: The identification of elevated BF oleic acid (a long-chain fatty acid) is consistent with recent microarray studies identifying altered lipid metabolism genes, while alterations in arginine and proline metabolism are consistent with recent studies identifying elevated L-arginine in DMD patient sera as a biomarker of disease. Together, these studies demonstrate muscle-specific alterations in GRMD-affected muscle, which illustrate previously unidentified metabolic changes.

## 1. Introduction

Duchene muscular dystrophy (DMD) is a neuromuscular disorder characterized by progressive loss of muscle mass that renders the mobility of its victims to wheelchair before the age of 12 [1]. Genetically, X-linked Duchenne muscular dystrophy is caused by the absence of dystrophin protein and is a lethal inherited disorder that mainly affects male. The disease progresses with a gradual development of respiratory insufficiency, cardiomyopathy, and skeletal muscle weakness, frequently causing death by the late teens or early twenties [2,3]. Of the three naturally-occurring mammalian (murine, feline, and canine) DMD models [4], in addition to the two non-mammalian (zebrafish and *Caenorhabditis elegans*) models [5], the canine models are thought to be the most suitable ones for studies because of their pathological resemblance and clinical relevance to human patients of DMD [5,6]. Like DMD, the genetically homologous Golden Retriever Muscular Dystrophy (GRMD) dog model of DMD is characterized by muscle necrosis, progressive paralysis, differential weakness of flexor and extensor muscles muscles, and mixed atrophy and hypertrophy of specific skeletal muscles [7,8]. The GRMD phenotype includes marked atrophy of the long digital extensor (LDE) as compared to sibling unaffected normal dogs, while the biceps femoris (BF), a member of the hamstring muscle group, undergoes less pronounced atrophy [7,8].

Recent studies have begun to implicate metabolic defects in muscular dystrophies, including DMD. Qualitative and quantitative lipid analysis of DMD, Becker’s Muscular Dystrophy, facioscapulohumeral muscular dystrophy (FSHD), and limb girdle muscular dystrophy-2B (LGMD-2B) patient biopsies found significant increases in glycogen [9]. Fatty acids were also altered in DMD, BMD, FSHD, and LGMD-2B, although DMD biopsies exhibited unique increases in cholesterol in DMD patients [9]. A recent microarray analysis of GRMD muscle identified the first evidence of potential alterations in metabolism, specifically alterations in genes associated with lipid metabolism and energy production [10]. Impairment of bioenergetics has recently been reported in congenital muscle dystrophy Type 1A (MDC1A) and Leigh Syndrome, linking metabolism to disturbances in skeletal muscle cell apoptosis [11]. In the present study, we undertook a non-targeted metabolomics analysis to determine how GRMD skeletal muscle compared to age-matched control muscle metabolically using gas chromatography-mass spectrometry (GC-MS) to identify underlying metabolic defects specific for GRMD skeletal muscle. 

## 2. Results

### 2.1. Determination of Metabolomics Changes in the GRMD Biceps Femoris vs. Control (t-Test)

**Figure 1:**
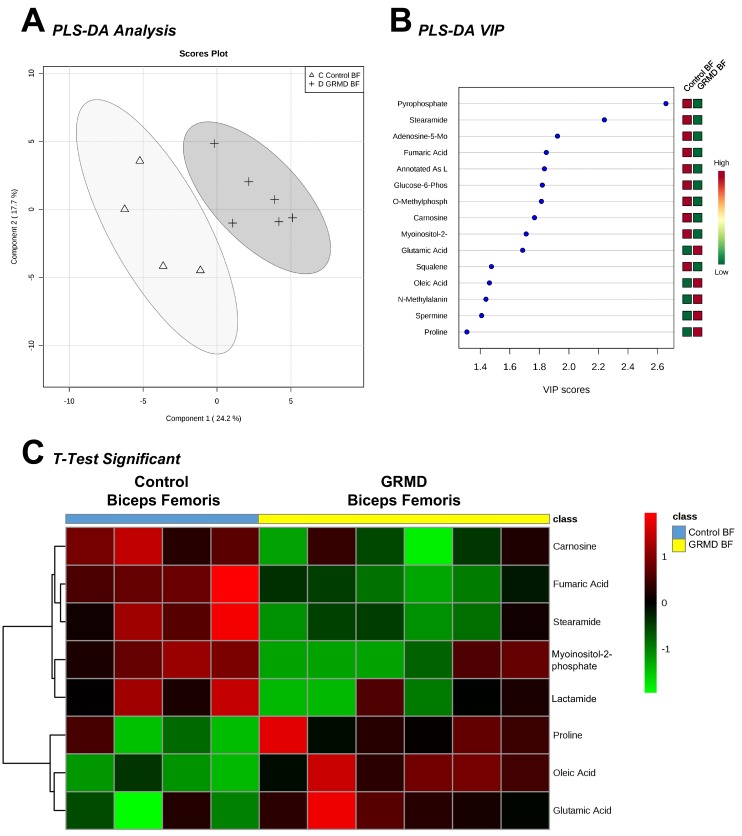
Untargeted metabolomics analysis of golden retriever muscular dystrophy (GRMD) biceps femoris (BF) muscle. (**A**) Supervised clustering of GRMD BF metabolites using Partial least squares discriminant analysis (PLS-DA); (**B**) The top metabolites ranked by VIP scores; (**C**) Heatmap of *t*-test significant metabolites identified in GRMD BF vs. age-matched controls. Analysis by Metaboanalyst analysis of GRMD (N = 6) vs. control (N = 4) BF metabolites.

The first statistical analysis we ran was designed to determine if there were significant alterations in the GRMD BF muscle compared to controls. To do this, we analyzed biopsy samples from GRMD-affected (and non-affected sibling) dogs at 6 months of age, at which time phenotypic changes have occurred [7,8]. Previous studies identified marked atrophy of the LDE compared to sibling unaffected normal dogs, while the BF, a member of the hamstring muscle group, undergoes less pronounced atrophy [7,8]. The limited number of samples run (6 GRMD and 4 controls) are due to the cost of housing and maintaining ill dogs for 6 months and the limited number of animals in the colony. A *t*-test was then performed on the metabolites to determine BF metabolites affected. Analysis of the less atrophied, early stage disease BF yielded 75 metabolites muscle (Figure S1A), including 19 imputed (Figure S1B), which upon partial least squares discriminant analysis (PLS-DA) produced separation of the control and GRMD groups from the first two components (Figure 1A). PLS-DA VIP analysis identified pyrophosphate (VIP Score 2.65) and stearamide (VIP Score 2.24) with the highest contributions to this signature (Figure 1B). *t*-Test analysis of the two groups identified eight significant metabolites (carnosine, fumaric acid, steramide, myoinositol-2-phosphate, lactamide, proline, oleic acid, glutamic acid, Figure 1C).

**Figure 2:**
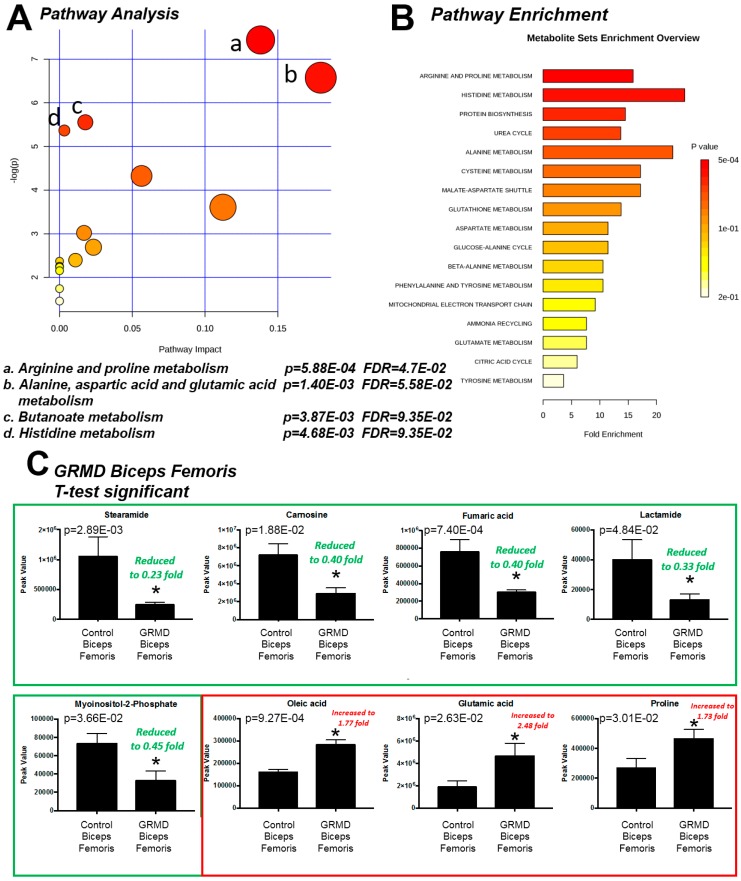
Pathway enrichment analysis of *t*-test significant metabolites from GRMD biceps femoris (BF) muscle. (**A**) Pathway analysis of *t*-test significant metabolites; (**B**) Enrichment analysis of *t*-test significant metabolites using pathway dataset for comparison; (**C**) Comparison of Peak values of *t*-test significant metabolites. Analysis by Metaboanalyst analysis of GRMD (N = 6) vs. control (N = 4) BF metabolites. Data is presented as the mean +/- SEM.

We next investigated what these eight significant metabolites had in common in terms of metabolic pathways. To do this, we determined their enrichment, or likelihood of being significantly altered by random chance, in metabolic pathways. Pathway analysis of these eight significant metabolites in GRMD BF identified four affected pathways, including: (1) arginine and proline metabolism; (2) alanine, aspartic acid, and glutamic acid metabolism; (3) butanoate metabolism, and (4) histidine metabolism, with false discovery rates (FDR) of 5–9% (Figure 2A). The FDR estimates the likelihood that a conclusion that a relationship exists, whereas in reality it does not for an individual comparison [12]. Its use in the the current context represents the likelihood that the pathways identified as significantly enriched may be false and is generally set at 5%. In the current studies, we have presented data with just higher FDR, as we are necessarily analyzing a smaller number of biological biopsies. Enrichment analysis of the BF *t*-test significant metabolites against a disease-associated metabolite set (blood) identified > 20-fold enrichment in histidine metabolism and alanine metabolism (Figure 2B). Enrichment of arginine and proline metabolism had the lowest p value (p = 5.88 × 10^−4^, FDR = 4.7 × 10^−2^) enriched > 15-fold (Figure 2B). To further identify metabolic pathways to which our significant metabolites may be related, we compared them to changes in other disease sets, including urine, CSF, and location-based sets for further clues to the metabolic pathways affected. Metabolic enrichment BF *t*-test significant metabolites were analyzed against disease-associated metabolite sets (urine, CSF) and location-based metabolite sets and identified nearly 80-fold enrichment for carnosinuria/carnosinemia (Figure S2A), > 10-fold for spinocerebellar degeneration (Figure S2B), and 5-fold for skeletal muscle (Figure S2C). Of the eight (8) *t*-test significant metabolites, five (5) were significantly decreased (stearamide, carnosine, fumaric acid, lactamide, and myoinositol-2-phosphate), while three (3) were significantly increased (oleic acid, glutamic acid, and proline) (Figure 2C).

### 2.2. GRMD vs. Control Long Digital Extensor Muscle vs. Control (t-Test)

**Figure 3:**
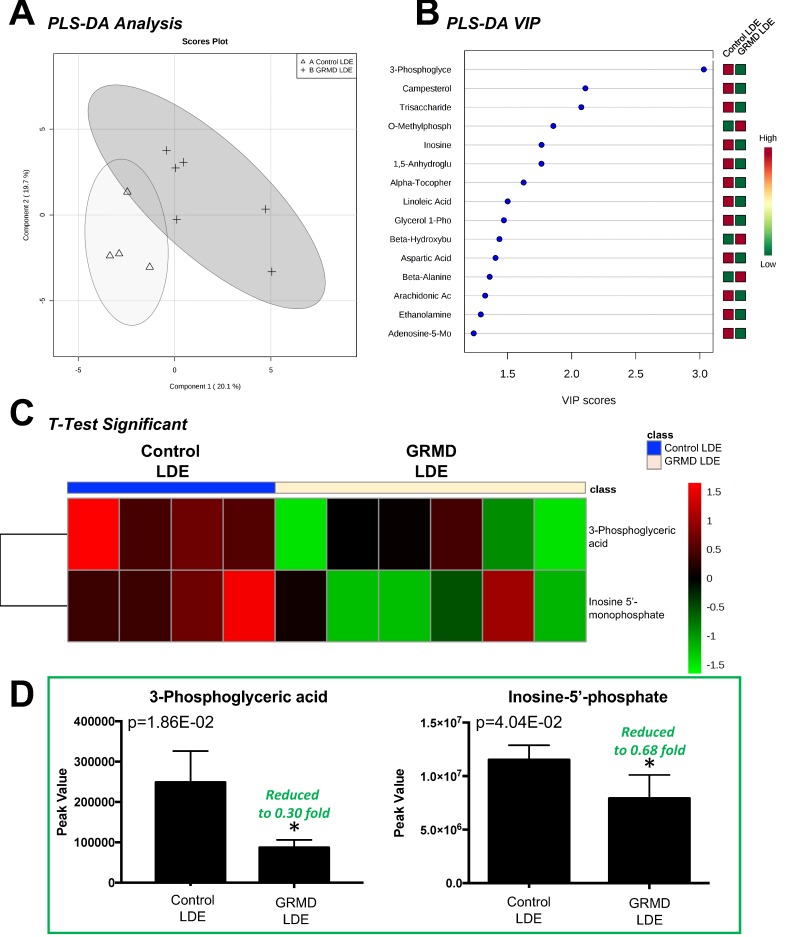
Untargeted metabolomics analysis of GRMD long digital extensor (LDE) muscle. (**A**) Supervised clustering of GRMD LDE metabolites using Partial least squares discriminant analysis (PLS-DA); (**B**) The top metabolites ranked by VIP scores; (**C**) Heatmap of *t*-test significant metabolites identified in GRMD BF vs. age-matched controls. Analysis by Metaboanalyst analysis of GRMD (N = 6) vs. control (N = 4) long digital extensor metabolites; (**D**) Peak values of significant metabolites identified in GRMD LDE vs. control LDE.

The second statistical analysis we ran was designed to determine if there were significant alterations in the more atrophied, later stage disease GRMD LDE compared to controls. The LDE functions to flex the tibiotarsal joint and also serves as a digital extensor [13] and is significantly atrophied in GRMD to a greater extent than the BF muscle [7,8]. In this analysis, we compared biopsy samples from GRMD-affected and non-affected sibling LDE samples at 6 months of age and performed a *t*-test to determine if there were any significant alterations in the GRMD LDE muscle compared to controls (Figure 3). Of the 75 metabolites identified in LDE muscles (Figure S3A), 14 were imputed (Figure S3B), separation on partial least squares discriminant analysis (PLS-DA) was seen (Figure 3A). PLS-DA VIP analysis identified inosine-5’-monophosphate (VIP Score 3.91) and 3-phosphoglyceric acid (VIP Score 3.08) with the highest contributions to this signature. *t*-Test analysis of the two groups identified two (2) significant metabolites (phosphoglyceric aid and inosine-5’-monophosphate (Figure 3C), which were both decreased (Figure 3D).

### 2.3. GRMD LDE vs. GRMD BF vs. Control LDE vs. Control BF (ANOVA)

**Figure 4:**
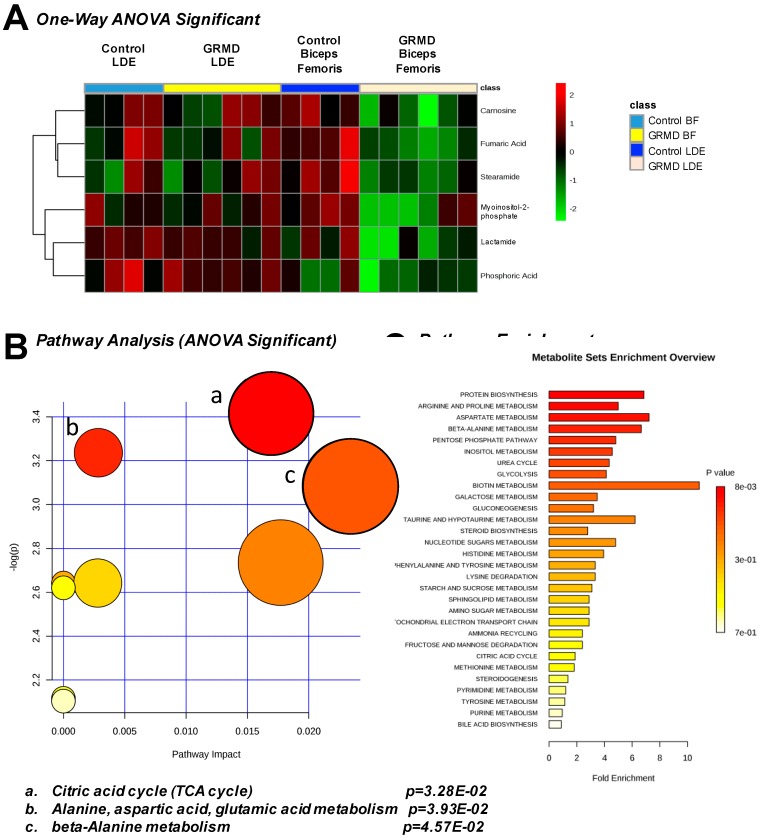
One-Way ANOVA analysis of GRMD long digital extensor (LDE) and biceps femoris (BF). (**A**) Heatmap of ANOVA significant metabolites from control and GRMD LDE and BF; (**B**) Pathway analysis of ANOVA significant metabolites; (**C**) Pathway analysis of ANOVA significant metabolites. Analysis by Metaboanalyst analysis of GRMD (N = 6) vs. control (N = 4) LDE metabolites.

**Figure 5:**
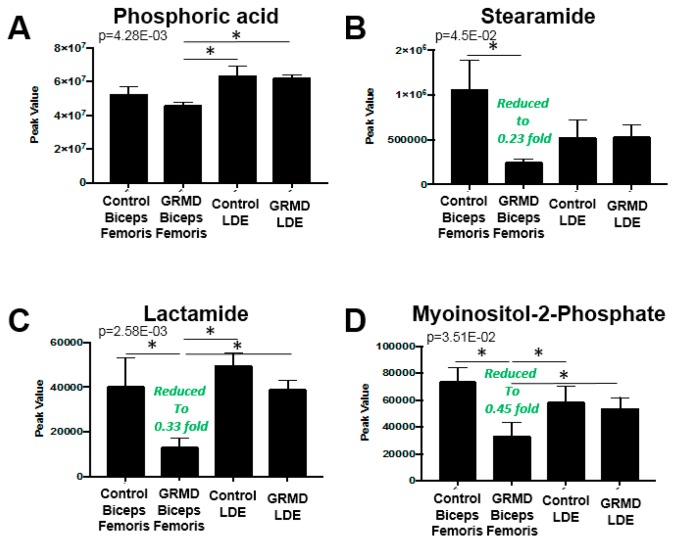
Comparison of Peak values of ANOVA metabolites in GRMD LDE and BF muscles by untargeted metabolomics. Peak values of GRMD LDE and BF (**A**) phosphoric acid; (**B**) stearamide; (**C**) lactamide; and (**D**) myosinositol-2-phosphate. Analysis by Metaboanalyst analysis of GRMD (N = 6) vs. control (N = 4) long digital extensor metabolites. Data is presented as the mean +/- SEM.

A third statistical analysis analysis was performed to identify if there were any common changes in the more atrophied later stage disase GRMD LDE and less atrophied, early stage GRMD BF compared to control LDE and BF dogs (four groups total). To do this, an ANOVA analysis was performed to identify any common changes in GRMD muscles, a question that the first two *t*-tests did not address (Figure 4). By PLS-DA analysis, considerable overlap of the BF metabolic profiles (GRMD and Control) and LDE (GRMD and Control) by both component 1 and component 2 was seen when all four groups were analyzed together (Figure S5). Of the 75 metabolites from each group (Figure S4), imputed as described above (Figure S1B, Figure S3B), eight (8) were significant, including carnosine, fumaric acid, stearamide, myoinositol-2-phosphate, lactamide, and phosphoric acid (Figure 4A). Pathway analysis identified three pathways as significant: (1) TCA cycle; (2) alanine, aspartic acid, glutamic acid metabolism; and (3) beta-alanine metabolism (Figure 4B), with > 6-fold enrichment for protein biosynthesis (Figure 4C). The unique significant metabolite in this analysis compared to the *t*-test run on the BF group was phosphoric acid, which was significantly less in the GRMD BF as compared to control and GRMD LDE groups (Figure 5A). While ANOVA analysis of stearamide revealed a single difference between control and GRMD BF muscles only (Figure 5B), the significant decreases in lactamide and myoinositol-2-phosphate seen in GRMD BF were significantly attenuated in GRMD LDE (Figure 5C,D).

**Figure 6:**
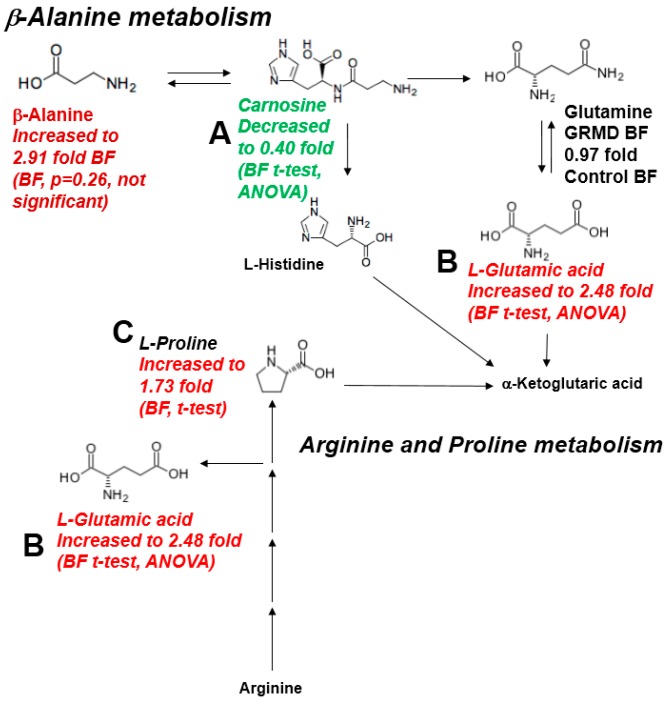
Significantly altered metabolites in the b-Alanine and Arginine/Proline metabolic pathways. (**A**) Carnosine decreased in BF by *t*-test and ANOVA; (**B**) Glutamic acid increased by in BF by *t*-test and ANOVA; (**C**) Proline increased in BF by *t*-test.

**Figure 7:**
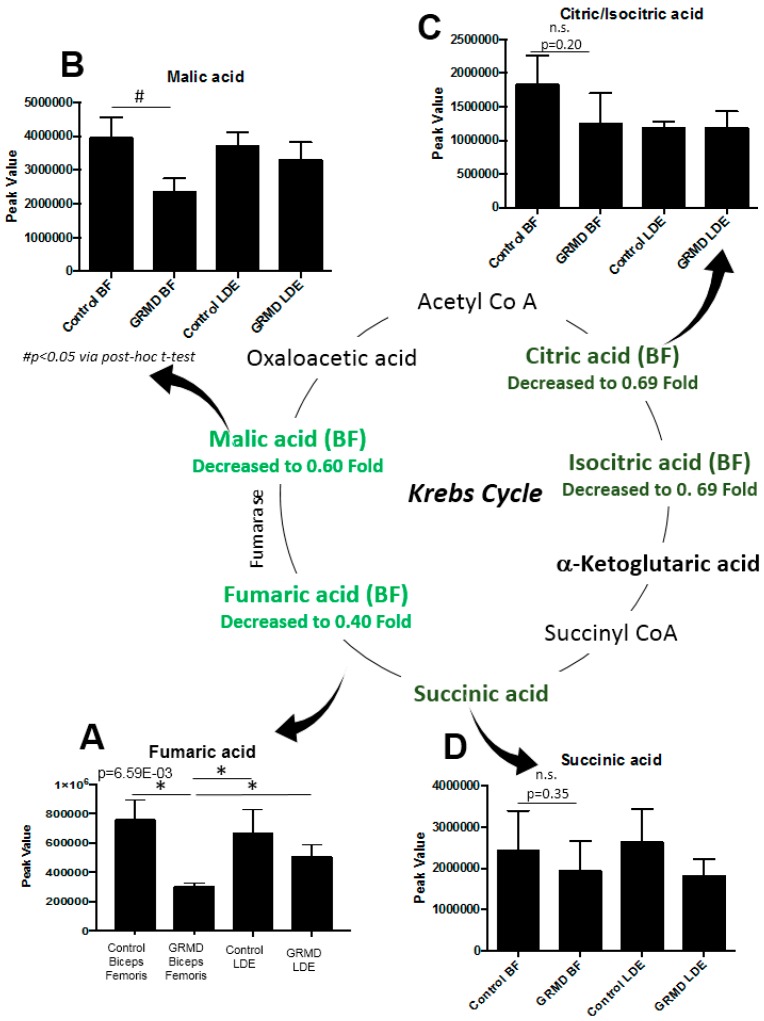
Significantly altered metabolites in the Krebs (TCA) Cycle in GRMD BF muscle by untargeted metabolomics. (**A**) Significantly decreased fumaric acid (One-Way ANOVA); (**B**) significantly decreased malic acid (*t*-test), with decreased (not significant by post-hoc *t*-test analysis); in (**C**) Citric/Isocitric acid; and (**D**) Succinic acid. Data is presented as the mean +/- SEM.

In the preceeding three analyses, pathway analyses identified that beta-alanine metabolism, arginine/proline metabolism, and the TCA cycle were the pathways most affected based on both the *t*-test and ANOVA significant metabolites identified in the GRMD BF (Figure 2A, Figure 4B). To further delineate the context of these alterations in context of their metabolic pathways, we next documented the changes in the pathways themselves (Figure 6). In the beta-alanine and arginine/proline metabolic pathways, significant decreases in carnosine were identified (Figure 6A), along with significant increases in glutamic acid and proline (Figure 6B,C). In BF, significant decreases in fumaric (Figure 7A) and malic acid (Figure 7B) were identified, with decreases in citric/isocitric acid (Figure 7C) and succinic acid also seen (not significant) (Figure 7D).

### 2.4. Integrated Metabolomics Analysis

The two primary approaches in functional analysis of metabolomics consist of metabolite set enrichment [14] and metabolic pathway analysis [15], as performed in the described in the prior two analyses [16]. A new approach that has been developed utilizes analysis of metabolomics experiments in combination with transcriptomics studies to exploit the models from KEGG (Kyoto Encyclopedia of Genes and Genomes) pathways using a method called integrated pathway analysis [15]. This integrated pathway analysis combining evidence from both metabolite concentrations and gene expression was developed to pinpoint pathways involved in the underlying biological processes [15]. In the present study, we performed an integrated pathway analysis using GRMD BF/LDE (6 months of age) significant metabolites caronosine, oleic acid, pyrophosphate, 3-phosphoglyceric acid, campesterol (Table S2) and 55 significantly altered genes from the GRMD gastrocnemius (6 months of age) (Table S3). This analysis was the first time that both metabolomics and microarray evidence were compared in combination, to determine how these complementary datasets mights point to common alterations in metabolism. 

**Figure 8:**
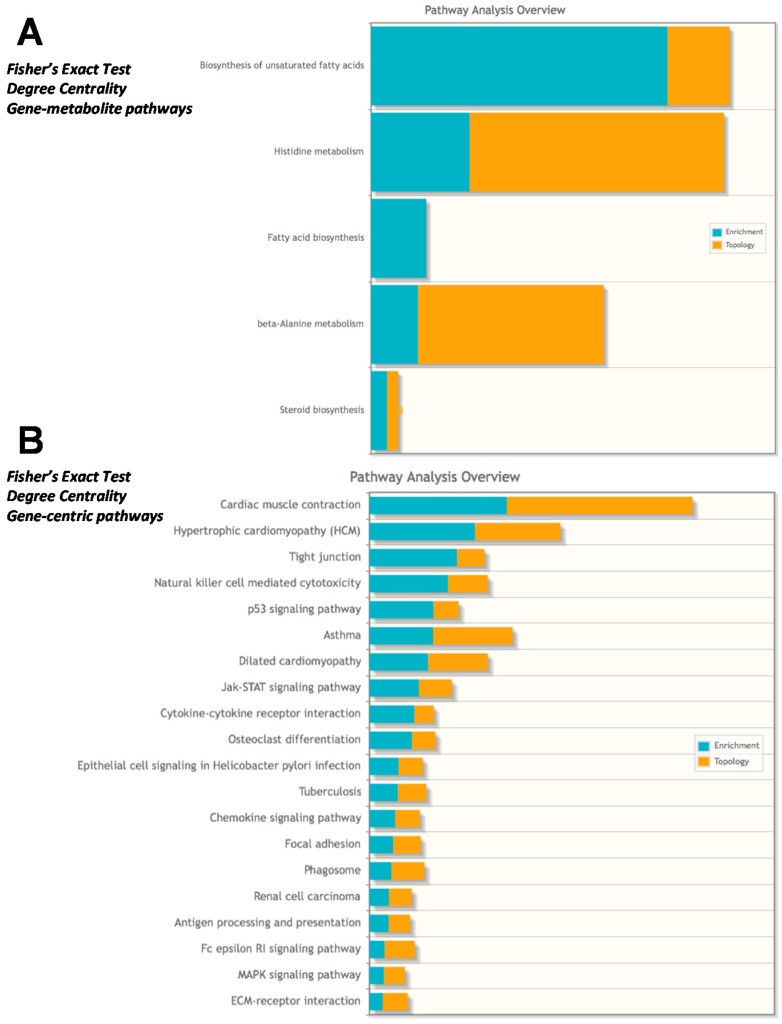
Integrated metabolomics analysis using recently published microarray analysis. Fisher’s exact test using degree centrality was performed using (**A)** Gene-metabolite pathways or (**B)** Gene-centric pathways in Metaboanalyst. GRMD significant metabolites (*t*-test, VIP >2.0 listed in Table S2) and mRNA >1.9 or < −1.3 fold from GRMD muscle (downloaded from GEO, as published in *Pediatr Res*. 2016 Apr;79(4):629-36) and listed in Table S3 with fold change calculations) were included in the Metaboanalyst integrated analysis.

The integrated pathway analysis gives two values for each metabolic pathway: enrichment and topology. Topology uses the structure of a given pathway to identify the relative importance of genes and compounds. In our first analysis, we analyzed the data sets to look at gene-metabolite pathways and found the most enriched/topology pathways to be “Biosynthesis of unsaturated fatty acids”, “Histidine metabolism”, and “β-Alanine metabolism” (Figure 8A). When the analysis was run in a gene-centric manner so that the transcriptomics were the main focus of analysis, we identified the most enriched/topology pathways to be “cardiac muscle contraction”, “hypertrophic cardiomyopathy”, and “tight junction” (Figure 8B). These findings expand upon the conclusions of the initial published microarray analysis of the GRMD heart and gastrocnemius tissues, finding a common alteration in lipid metabolism, energy production, and inflammatory responses [10] and the primary finding that the highest induced transcript in the GRMD gastrocnemius muscle was SPP1 [10], previously shown to be an indicator of muscle injury prior to microarray analyses [17]. 

## 3. Discussion

The metabolic component of DMD has not been previously analyzed directly. At the transcriptional level, studies of GRMD skeletal, but not cardiac, muscle have been described as consistent with a “metabolic crisis” [18], with the down-regulation of dozens of energy production-associated mRNAs and multiple electron transport component mRNAs in the TCA cycle [18]. In the present study, we investigated two GRMD pelvic limb muscles, the more proximal BF muscle which is a member of the hamstring group and is relatively spared and the more distal LDE that undergoes progressive atrophy. We hypothesized that the BF and LDE would have different metabolic profiles consistent with their milder/earlier versus more severe/chronic stages of disease, respectively. There were several differences in metabolism, with nine significantly altered metabolites in the GRMD BF muscle (eight by *t*-test, Figure 2C, one additional by ANOVA, Figure 4A) as compared to only 2 in the more chronically affected GRMD muscle (LDE). In addition, we identified for the first time in this system significant increases in metabolites involved in β-alanine metabolism and arginine/proline metabolism (Figure 6).

There are non-specific conditions that have been linked to muscle catabolism in humans, which may lower muscle glutamic acid levels, including emphysema [19], COPD [20], elderly fraility [21], and in acute sepsis and surgical trauma [19]. Several studies have been suggestive of these metabolic changes in DMD, using NMR analyses. In the mdx mouse, recent studies using ^1^H-magnetic resonance spectroscopy to study the metabolic profile of quadriceps and diaphragm muscle identified increases in glutamic acid and carnosine [22]. Other studies using NMR analysis of vastus lateralis muscle biopsies from DMD patients (N = 11 vs. 7 normal controls) identified significant decreases in glutamate/glutamine (not discernable from each other) [23]. 

Skeletal muscles most abundantly require glutamic acid [24]. Glutamic acid is the only amino acid that is actively taken up from the circulation by the muscles in the post-absorptive state [25]. Intracellular degradation of myofibrillar proteins also release glutamic acid with large amounts contributing to the muscle glutamic acid pool [26]. The role of glutamic acid in muscular dystrophy was studied in the early 1960s in rabbit models (due to vitamin E deprivation) [27]. In these studies, body weight loss and the onset of terminal disease was accelerated when animals were given exogenous glutamic acid [27]. While the reasons for and sources of elevated BF glutamic acid levels (~2.5-fold controls) are not clear (Figure 6), their presence could potentially play a role in the eventual worsening of the disease process.

The role of oleic acid in DMD disease has been investigated in the mdx mouse model, where dietary oleic acid was replaced with the n-3 poly-unsaturated fatty acid (PUFA), alpha-linolenic acid (ALA) [28]. Interestingly, these studies found that shifting to a high PUFA diet resulted in the expected higher n-3 PUFA content in muscle, while reducing arachidonic acid content in skeletal muscle phospholipids, but also made the mdx muscle more susceptible to sarcolemmal leakiness [28]. When DMD patient erythrocytes have been studied for long chain fatty acid composition, decreases in oleic, linoleic, and arachidonic acids have been identified [29]. Where these fatty acids are being utilized, however, has not been investigated, nor have their levels in skeletal muscle been determined, to our knowledge. In the present study, the significantly elevated oleic acid (18:0) in GRMD BF muscle (Figure 2C) may represent alternate energy substrate utilization and be directly related to the decreased oleic acid (18:0) metabolite stearamide (Figure 2C). Alternatively, the significant increase and GRMD BF oleic acid may be a consequence of a greater accumulation of muscle triacylglyceride (TAG), a storage form of oleic acid. In the GRMD model, fatty infiltration is rare before 4 months of age, but common in those > 13 months of age [8], so would be expected to be present in the 6 month old muscle investigated here. If this is the case, its possible that mitochondrial dysfunction may be responsible, as mitochondrial dysfunction has been reported to induce lipid droplet formation (including triacyglycerol and cholesterol esters) in reponse to stress [30].

The primary amides of oleic (18:0), palmitic, palmitoleic, eladic, and linoleic acid were identified in human plasma in 1989, although their significance was not understood [31]. Subsequently, other fatty acid amides (including stearamide) have been isolated from human tear gland secretions [32]. The role of fatty acid amides is not completely clear, but the fact that the arachidonic acid metabolite arachidonamide is the best substrate for the membrane-bound serine hydrolase fatty acid amide hydrolase (FAAH) suggests a role in signaling [33]. In muscle, primary fatty acid amides reduced sarco/endoplasmic reticulum Ca^2+^-ATPase (SERCA) activity in the micromolar (μM) range, although stearamide inhibition was relatively minimal [34]. The significance of the reduced GRMD BF stearamide (0.2-fold control muscle) is not clear but may reflect altered signaling pathways within affected skeletal muscle not seen in unaffected GRMD muscle (LDE, Figure 5B).

Carnosine (β-alanyl-L-histidine) is a dipeptide found to be highly concentrated in skeletal muscles [35]. At least three roles have been attributed to carnosine, including: a lactic acid neutralizer under anaerobic conditions [36], a potent scavenger of singlet oxygen protecting the cells from oxidative damage [37], and a physiological activator of myofibrillar ATPase activity [38]. The significant decrease in GRMD BF carnosine (0.4-fold control BF levels, Figure 2C) could set the stage for eventual muscle damage (e.g. due to lactic acid or oxidative damage). It may also be a cause of limited myosin ATPase activity, which relies on carnosine [39], and be a contributing factor to muscle fatigue. It has been reported that vitamin B6 plays a pivotal role in maintaining the carnosine concentrations in the skeletal muscles [40,41]; however, vitamin B6 (pyridoxine) was not measured in the present study in order to determine if this was an underlying cause. Interestingly, GRMD BF β-alanine levels were significantly increased (2.9-fold of control levels), while carnosine was decreased (Figure 6). Since carnosine is hydrolyzed to its constituent amino acids (β-alanine and histidine) by the enzyme carnosinase [42], increased carnosinase may be a contributing cause of the elevated GRMD β-alanine and decreased carnosine.

In the present study, we identified elevated proline and factors associated with the arginine/proline metabolic pathway. Arginine is taken up by cells, metabolized to proline, which can be used as a precursor for collagen [43]. This is significant because a major finding in chronic myopathies, such as DMD, is collagen deposition/fibrosis. Previous studies of the GRMD cranial sartorius muscle identified patchy increases in endomysial space as early as 6 months of age [8]. In previous studies, the GRMD dog muscles were found to have higher levels of hydroxyproline, a biomarker of fibrosis and collagen content, across muscles (cranial sartorius, vastus lateralis, long digital extensor, and lateral head of the gastrocnemius) compared to controls at 8–9 months of age [44]. Similarly, increased fibrosis has been reported in 12 month old GRMD carrier hearts, along with inflammation and/or fatty changes, compared to control hearts [45]. Since elevated levels of proline are detected in the pathophysiology of fibrosis, including liver fibrosis [46], sarcoidosis [47], and rheumatoid arthritis [47,48], the increases in the GRMD proline/arginine metabolic pathway could be linked to increases in inflammation-related collagen synthesis/muscle fibrosis known to the be a central part of the pathogensis of DMD.

There are weaknesses and assumptions in the present study to consider. These include the limited number of biological replicates analyzed per group, due to the expense and rarity of the GRMD model itself. In particular, the identification of metabolites that were decreased, but not significant (e.g., citric/isocitric acid, and succinic acid, Figure 7) may reflect either a lack of samples (i.e., power), the variability of the phenotype, or may actually not be different. Therefore, caution should be exercised in interpreting these data and the findings should not be used as evidence to support a hypothesis. Additionally, the data presented here may be a consequence of the observed muscular dysfunction, and not a cause of the effects on the TCA cycle intermediate pools. For example, brief periods of endurance training can reduce TCA intermediate pools, as does overweight-to-obese sedentary lifestyles [49], whereas dynamic exercise can increase TCA intermediates [50] in the absence of disease. 

### Summary

While transcriptional analysis of GRMD skeletal muscle has suggested a metabolic component by the down-regulation of dozens of energy production-associated mRNAs and multiple electron transport component mRNAs in the TCA cycle, the present study is the first to our knowledge to analyze the metabolic component directly. We utilized untargeted metabolomics analysis to assess two GRMD pelvic limb muscles, the more proximal BF muscle which is a member of the hamstring group and is relatively spared, and the more distal LDE that undergoes progressive atrophy. We hypothesized that the BF and LDE would have diffent metabolic profiles consistent with their milder/earlier versus more severe/chronic stages of disease, respectively. There were eight (8) significantly altered metabolites in the milder/earlier stage disease GRMD BF muscle, including five (5) which were decreased (stearamide, carnosine, fumaric acid, lactamide, and myoinositol-2-phosphate) and three (3) which were increased (oleic acid, glutamic acid, and proline). Pathway analysis of these metabolites identified enrichment for arginine/proline metabolism and β-alanine metabolism. Identification of alterations across all four groups (GRMD BF, Control BF, GRMD LDE, Control BF) additionally identified phosphoric acid. Overall, many Krebs cycle intermediates were significantly decreased (e.g., malic acid, fumaric acid) and other intermediates were decreased but not significant (e.g., citric/isocitric acid, and succinic acid). These findings support the first direct evidence for decreases in TCA cycle intermediates and suggest a mechanism of altered energy metabolism may be underlying the observed dysfunction. We also identified elevated levels of the monounsaturated fatty acid oleic acid (18:0) in GRMD BF muscle, which has been related to increased inflammation in DMD that would be expected in earlier stages of disease, with associated necrosis. 

## 4. Materials and Methods 

### 4.1. Golden Retriever Muscular Dystrophy Dog Model

All GRMD dogs used in this study were from a colony at the University of North Carolina at Chapel Hill (UNC-CH), now located at Texas A&M University. Dogs were produced by mating a heterozygote affected male to a carrier female, such that ~ 25% each of the pups should be either normal males, affected males, heterozygous (carrier) females, or homozygous (affected) females. Normal females are not produced with this mating. Blood creatine kinase levels taken shortly after birth were used to diagnose neonates with GRMD [51], along with PCR, as previously described [52]. Samples of the BF and LDE muscles were taken at necropsy from GRMD (n = 6; 4 males and 2 females) and littermate wild type (control) (n = 4; all male) dogs at 6 months of age immediately following barbiturate euthanasia. These dogs were part of a larger cohort that was studied previously with a genome wide association study (GWAS) [53]. Dogs were used and cared for according to principles outlined in the National Research Council Guide for the Care and Use of Laboratory Animals. The experiments were approved by the Institutional Animal Care and Use Committee (IACUC) review board at UNC-CH and performed in accordance with federal guidelines.

### 4.2. Metabolomics Determination by GC–MS Instrumentation

Bicep femoris (BF) and long digital extensor (LDE) muscles were flash frozen after biopsy in liquid nitrogen and stored at –80 °C. A fraction of the muscle was then weighed (~25–50 mg wet weight), finely minced, and quickly added to fresh ice-cold pre-made buffer (50% acetonitrile, 50% water, 0.3% formic acid) at a standard concentration of 25 mg/475 μL, homogenized on ice for 20–25 seconds and stored at −80 °C. The samples were “crash” deproteinized by methanol precipitation and then spiked with D27-deuterated myristic acid (D27-C14:0) as an internal standard for retention-time locking only and dried. The deuterated standard was not used for relative quantification/correction of analyte peak responses due to the limited number of samples processed in single batches for the different tissues. The derivatized trimethylsilyl (TMS)-D27-C14:0 standard retention time (RT) was set at ~16.727 min. Reactive carbonyls were stabilized at 50 °C with methoxyamine hydrochloride in dry pyridine. Metabolites were made volatile with TMS groups using N-methyl-N (trimethylsilyl) trifluoroacetamide or MSTFA with catalytic trimethylchlorosilane at 50 °C. GC/MS methods generally follow those of Roessner et al., [54], Fiehn et al., [55], and Kind et al., [56], and used a 6890N GC connected to a 5975B inert single-quadrupole MS (Agilent Technologies, Santa Clara, CA, USA). The two wall-coated, open-tubular (WCOT) GC columns connected in series were both from J&W/Agilent (part 122–5512), DB5-MS, 15 meters in length, 0.25 mm in diameter, with an 0.25 μm luminal film. Positive ions generated with conventional electron-ionization (EI) at 70 eV were scanned broadly from 600 to 50 m/z in the detector throughout the 45-min cycle time.

Data were acquired using MSD ChemStation (Agilent Technologies) and metabolites annotated based on their mass fragmentation patterns and RT. Raw data formatted files were exported for further analysis in Automatic Mass Spectral Deconvolution and Identification Software (AMDIS version 2.72, build 140.24, freeware developed by Steve Stein, W. Gary Mallard, and their coworkers at National Institute of Standards and Technology or NIST [57,58,59]). Deconvoluted spectra were annotated as metabolites, to the extent possible, using an orthogonal approach that incorporates both RT from GC and the fragmentation pattern observed in EI-MS. Peak annotation is based primarily on our own RT-locked spectral library of metabolites. The library is built upon the Fiehn GC/MS Metabolomics RTL Library (a gift from Agilent, part number G1676-90000), Golm Metabolome Library [56] (courtesy of Joachim Kopka and coworkers at the Max Planck Institute of Molecular Plant Physiology, Golm, Germany [60]), the Wiley 9th-NIST 2011 commercial library (Agilent G1730-64000), and other spectral libraries. Once annotation was complete, a cross-tabulated spreadsheet was created, listing the integrated peak area for each metabolite versus sample identity. This was accomplished using a custom Visual Basic program in Microsoft Excel that grouped peaks across samples based on identical metabolite annotation and RT proximity. Peak alignment across samples was further confirmed using SpectConnect [61] to assess similarity in spectral fragmentation patterns and by manual curation. The raw, transformed, and sorted data used for each of the three comparisons in the metabolomic analyses (next) can be found in Table S1. The data obtained in this study is accessible at the NIH Common Fund’s Data Repository and Coordinating Center (supported by NIH grant, U01-DK097430) website, http://www.metabolomicsworkbench.org.

### 4.3. Metabolomic Statistical Analysis

Metaboanalyst (v3.0) run on the statistical package R (v2.14.0) used metabolite peak areas (as representative of concentration) [16,62,63,64]. The control group contained 4 biological replicates, while the GRMD group contained 6. If more than one individual in the control group did not have a metabolite detected in a group (of 4 total), that metabolite was excluded from further analysis. Likewise, if more than 2 individuals in the GRMD group did not have a metabolite detected in a group (of 6 total), that metabolite was excluded. In groups with values missing, the lowest value of that group was used to impute those values. These data were scaled using the Pareto scaling feature. Initially, a *t*-test comparing control to GRMD muscles was performed on both the BF and LDE muscle. In a second analysis, a One-Way Analysis of Variance (ANOVA) and Fisher LSD post-hoc test across the groups (control BF, GRMD BF, control LDE, GRMD LDE) was performed using Metaboanalyst v3.0. The data were analyzed by both unsupervised principal component analysis (PCA) and supervised partial least squares discriminant analysis (PLS-DA) to further determine the metabolites that separated groups. The specific metabolites contributing most significantly to the differences identified by PLS-DA between control BF and GRMD BF (or control LDE and GRMD LDE) muscles were determined using the variable importance in projection (VIP) analysis in the Metaboanalyst environment. Both *t*-test and ANOVA significant metabolites were matched to metabolomics pathways using the Pathway Analysis feature in Metaboanalyst 3.0. Only metabolites identified in the one-way ANOVA were included. All data from this study are available in Table S1. All heat maps were generated using the heat map analysis feature in Metaboanalyst v3.0. Data are presented as mean +/- SEM, unless otherwise indicated.

### 4.4. Integrated Microarray and Metabolomics Statistical Analysis

The metabolites used in the integrated microarray analysis included *t*-test significant and VIP > 2 metabolites from BF and LDE, limited to those with HMDB designations (carnosine, oleic acid, pyrophosphate, 3-phosphoglyceric acid, and campesterol) (Table S2). The microarry datasets used in parallel were accessed from GEO (GSE68626) and extracted using the GEO2R online tool, which were recently published [10]. Briefly, datasets from skeletal muscle (gastrocnemius) tissue from normal golden retriever (or golden retriever muscular dystrophy model) as were used in the current analysis. The normal golden retriever medial head of the gastrocnemius muscles (N = 3) were from animals ages 6, 6, and 7.5 months (accession number GSM1677427, GSM1677428, GSM1677429, respectively). The GRMD gastrocnemius medial head of the gastrocnemius muscles (N = 3) were from animals ages 6.75, 6, and 6 months (GSM1677430, GSM1677431, GSM1677432, respsectively). Data included GRMD mRNA expressed > 1.9 fold (vs. control) and < −1.3 fold (found in Table S3).

### 4.5. Other Statistical Analysis 

Metabolites not found to be significant were analyzed in a post-hoc analysis by a Student’s *t*-test in Microsoft Excel (Version 15.34 (170515, Office 365, Seattle, WA, USA) using a one-tailed test assuming the two-samples had heteroscedastic (unequal) variance and plotted in Prism 7.0 (GraphPad Software, Inc., La Jolla, CA, USA).

## Supplementary Materials

The following are available online at www.mdpi.com/2218-1989/7/3/38/s1, Figure S1: Heatmap analysis of all named metabolites from GRMD and control biceps femoris (BF) included in the t-test analysis. N = 4 control BF and N = 6 GRMD BF, Figure S2: Pathway analysis of GRMD biceps femoris t-test significant metabolites, Figure S3: Heatmap analysis of all named metabolites from GRMD and control long digital extensor (LDE) muscle included in the t-test analysis, Figure S4: Heatmap analysis of all named metabolites from GRMD and control long digital extensor (LDE) and biceps femoris (BF) muscle included in the One-Way ANOVA analysis, Figure S5: Untargeted metabolomics analysis of golden retriever muscular dystrophy (GRMD) biceps femoris (BF) and long digital extensor (LDE) muscle, Table S1: Non-targeted metabolomics performed on six month old control and age-matched golden retriever muscular dystrophy (a) biceps femoris used in t-test analysi; (b) long digital extensor used in t-test analysis; (c) long digital extensor or biceps femoris used in ANOVA analysis, Table S2: Significant metabolites included in combined gene and metabolite analysis in MetaboAnalyst3.0, Table S3: T-test Significant mRNA in GRMD by Microarray used in the combined gene and metabolite analysis in MetaboAnalyst 3.0.

## Abbreviations

The following abbreviations are used in this manuscript:

BF	biceps femoris
DMD	Duchenne muscular dystrophy
FDR	false discovery rate
GRMD	golden retriever muscular dystrophy
LDE	long digital extensor
