# Supplementary material for: Non-Targeted Metabolomics Analysis of Golden Retriever Muscular Dystrophy-Affected Muscles Reveals Alterations in Arginine and Proline Metabolism, and Elevations in Glutamic and Oleic Acid In Vivo
Source: Metabolites. 2017 Jul 29;7(3):38. doi: 10.3390/metabo7030038 (PMC5618323; doi:10.3390/metabo7030038)
Supplement: Supplementary file 1 [file metabolites-07-00038-s001.zip › metabolites-205630-publish-supplement/Supplement Figures.pptx]

## Slide 1
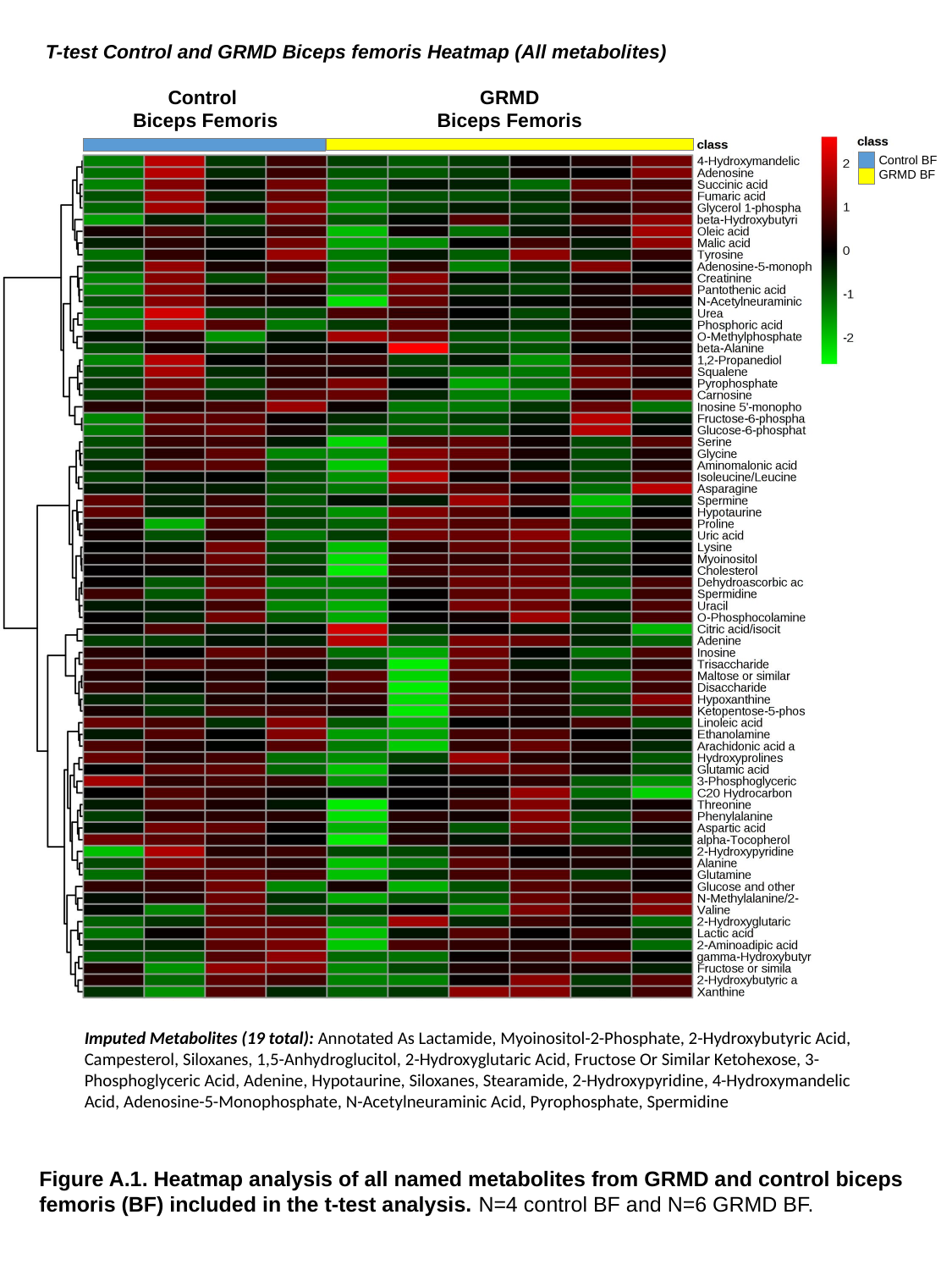

T-test Control and GRMD Biceps femoris Heatmap (All metabolites)
Control
Biceps Femoris
GRMD
Biceps Femoris
Control BF
GRMD BF
Imputed Metabolites (19 total): Annotated As Lactamide, Myoinositol-2-Phosphate, 2-Hydroxybutyric Acid, Campesterol, Siloxanes, 1,5-Anhydroglucitol, 2-Hydroxyglutaric Acid, Fructose Or Similar Ketohexose, 3-Phosphoglyceric Acid, Adenine, Hypotaurine, Siloxanes, Stearamide, 2-Hydroxypyridine, 4-Hydroxymandelic Acid, Adenosine-5-Monophosphate, N-Acetylneuraminic Acid, Pyrophosphate, Spermidine
Figure A.1. Heatmap analysis of all named metabolites from GRMD and control biceps femoris (BF) included in the t-test analysis. N=4 control BF and N=6 GRMD BF.

## Slide 2
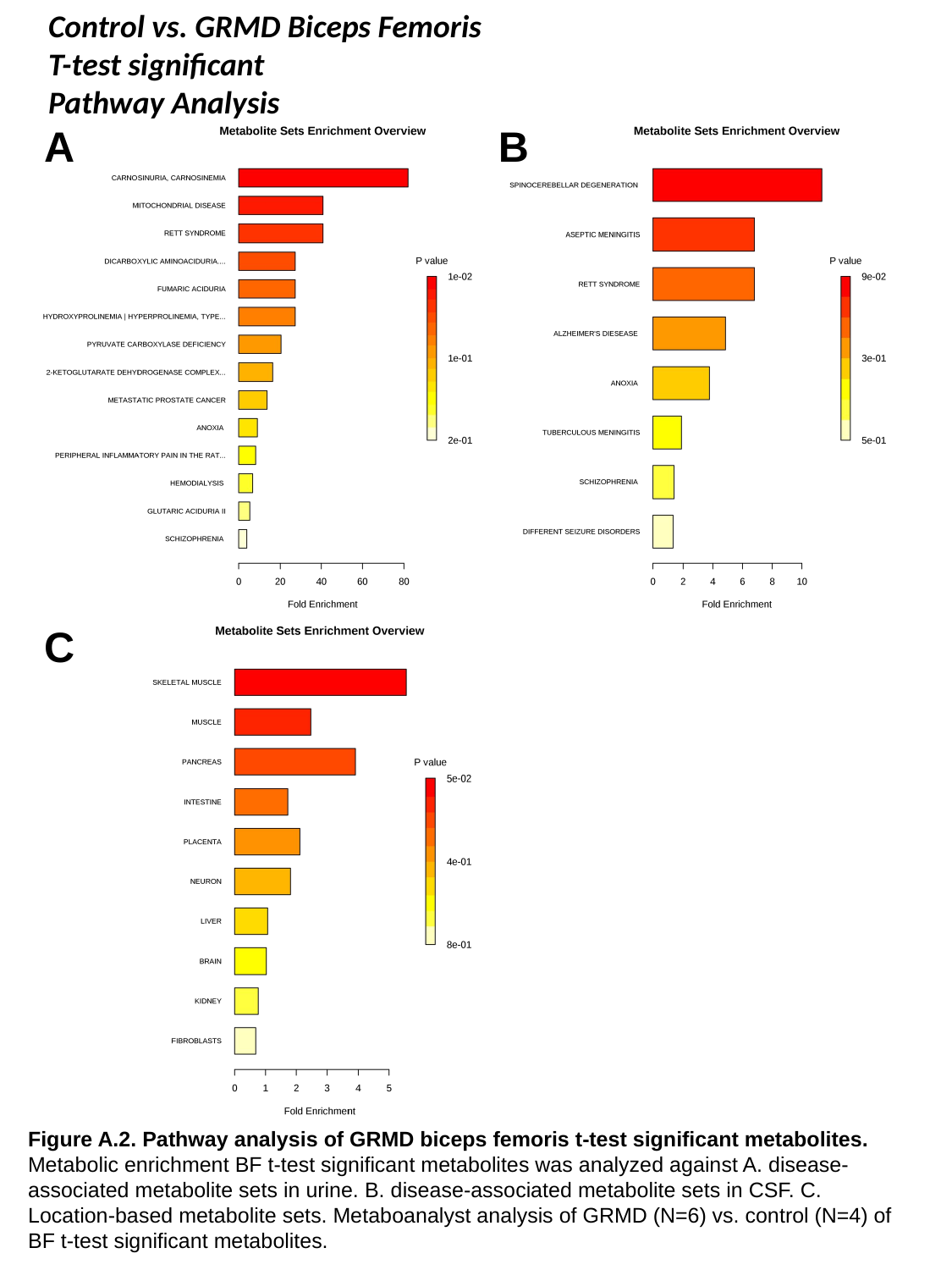

Control vs. GRMD Biceps Femoris
T-test significant
Pathway Analysis
A
B
C
Figure A.2. Pathway analysis of GRMD biceps femoris t-test significant metabolites. Metabolic enrichment BF t-test significant metabolites was analyzed against A. disease-associated metabolite sets in urine. B. disease-associated metabolite sets in CSF. C. Location-based metabolite sets. Metaboanalyst analysis of GRMD (N=6) vs. control (N=4) of BF t-test significant metabolites.

## Slide 3
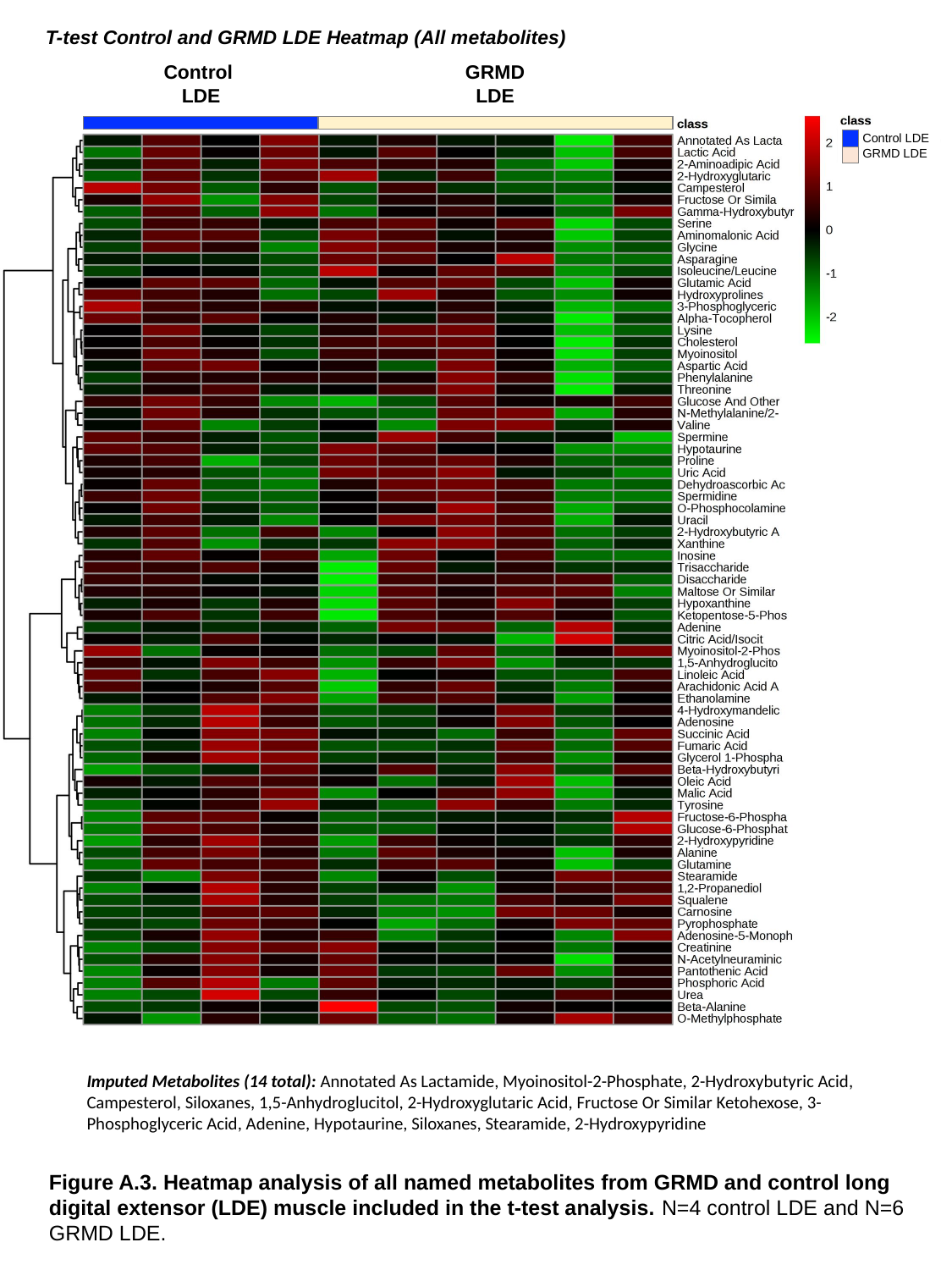

T-test Control and GRMD LDE Heatmap (All metabolites)
Control
LDE
GRMD
LDE
Control LDE
GRMD LDE
Imputed Metabolites (14 total): Annotated As Lactamide, Myoinositol-2-Phosphate, 2-Hydroxybutyric Acid, Campesterol, Siloxanes, 1,5-Anhydroglucitol, 2-Hydroxyglutaric Acid, Fructose Or Similar Ketohexose, 3-Phosphoglyceric Acid, Adenine, Hypotaurine, Siloxanes, Stearamide, 2-Hydroxypyridine
Figure A.3. Heatmap analysis of all named metabolites from GRMD and control long digital extensor (LDE) muscle included in the t-test analysis. N=4 control LDE and N=6 GRMD LDE.

## Slide 4
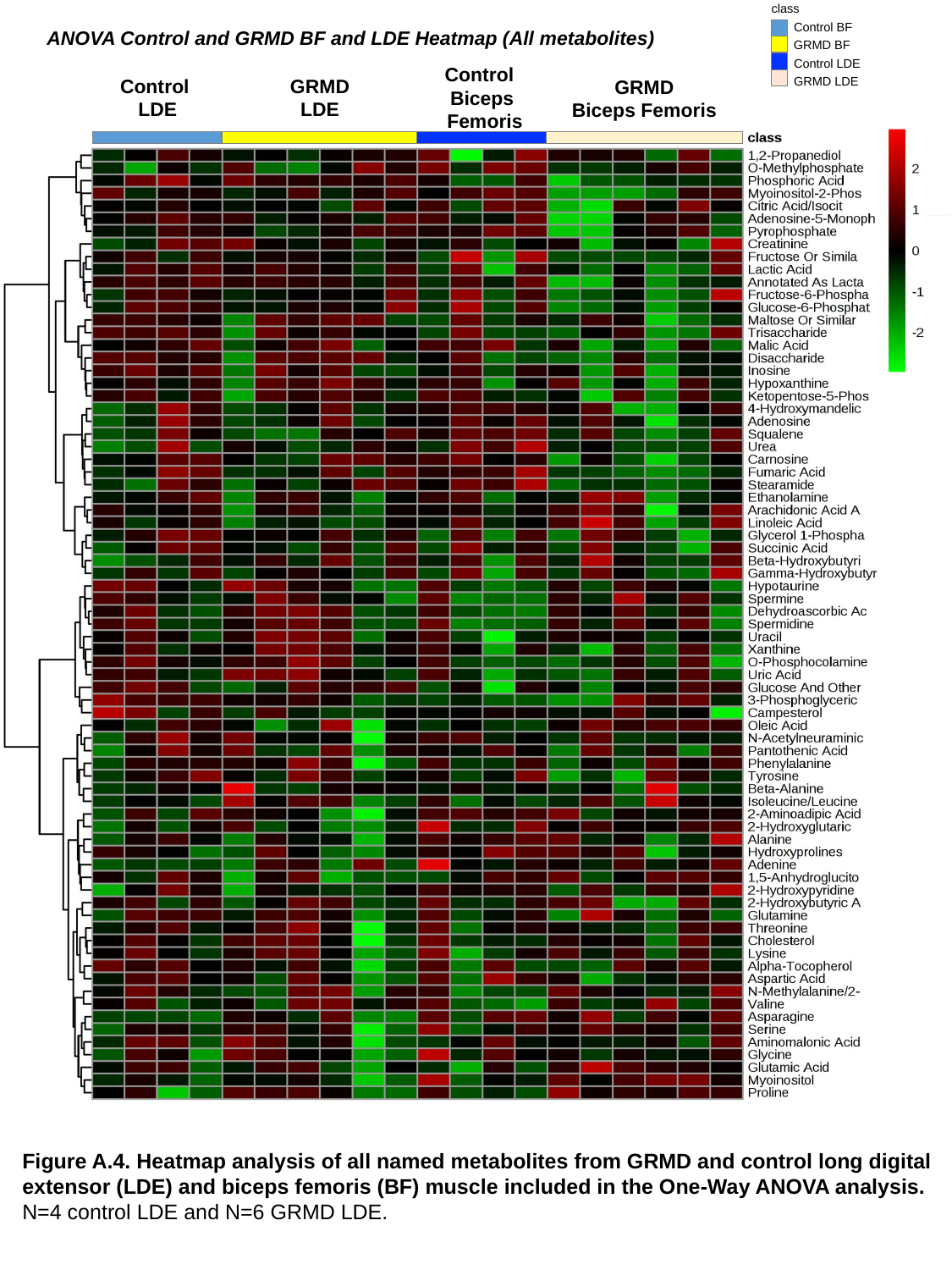

class
Control BF
GRMD BF
Control LDE
GRMD LDE
ANOVA Control and GRMD BF and LDE Heatmap (All metabolites)
Control
Biceps
 Femoris
Control
LDE
GRMD
LDE
GRMD
Biceps Femoris
Figure A.4. Heatmap analysis of all named metabolites from GRMD and control long digital extensor (LDE) and biceps femoris (BF) muscle included in the One-Way ANOVA analysis. N=4 control LDE and N=6 GRMD LDE.

## Slide 5
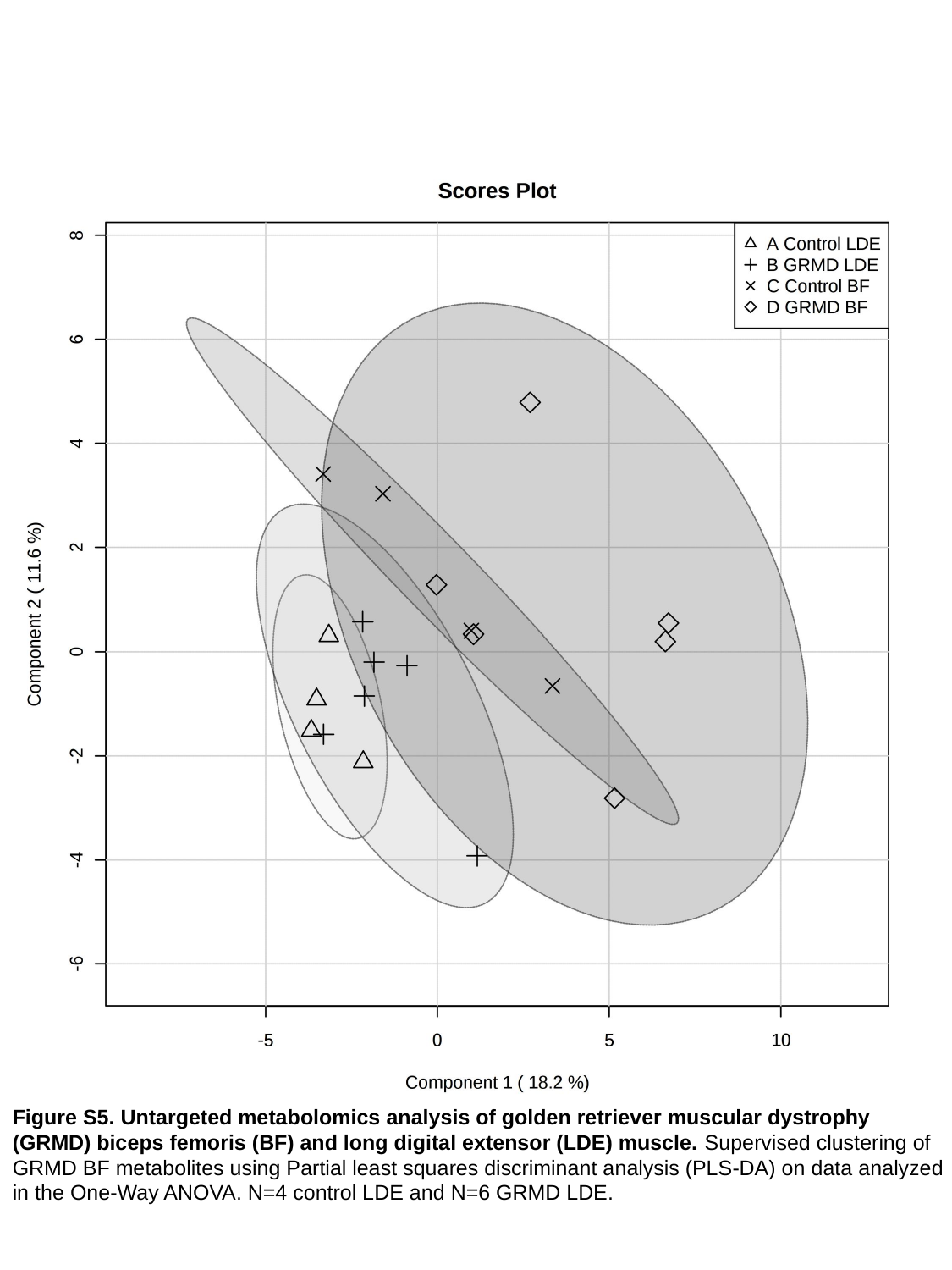

Figure S5. Untargeted metabolomics analysis of golden retriever muscular dystrophy (GRMD) biceps femoris (BF) and long digital extensor (LDE) muscle. Supervised clustering of GRMD BF metabolites using Partial least squares discriminant analysis (PLS-DA) on data analyzed in the One-Way ANOVA. N=4 control LDE and N=6 GRMD LDE.
